# The impact of anthropogenic organic and inorganic pollutants on the Hasdeo River Water Quality in Korba Region, Chhattisgarh, India

**DOI:** 10.6026/97320630016332

**Published:** 2020-04-30

**Authors:** Monika Bhaskar, Ashwini Kumar Dixit, Kundan Kumar Ojha, Satish Dubey, Akanksha Singh, Amar Abhishek

**Affiliations:** 1 School of Life Sciences, Department of Botany, Guru Ghasidas Vishwavidyalaya, Bilaspur-495009, Chhattisgarh, India; 2 King George's Medical University, Lucknow, Uttar Pradesh- 226003, India

**Keywords:** Hasdeo River, heavy metals, water quality index (WQI), heavy metal pollution index (HPI), metal index (MI)

## Abstract

In the name of development, industries discharge their wastewater, which contains different Metallic species and massive organic load into the next-door river system. In this study, we assess the impact of organic and inorganic contaminations on Hasdeo River at Korba region, which is fifth critically polluted city in India. Hear, a new approach for water quality indexing like Water quality index (WQI), Heavy metal pollution index (HPI) and metal index (MI) has been proposed to represent pollution due to heavy metals in river system. The sample's pollution parameters and heavy metals contamination is exceed from BIS or WHO standards of drinking water (all p<0.05). WQI shows that the entire water samples are not suitable for drinking and aquatic life but they are safe only for irrigation. HPI and MI calculation revels that more than 95% sampling sites are critically polluted with heavy metals. Thus, a high level of industrialization deterioration of river water quality is recorded for adequate action.

## Background

Rivers are the most important fresh water resource in the world and supply water for many purposes. India has been blessed huge amount of surface water in the form of rivers. In the name of development, Industries discharge their treated or partially treated wastewater, which contains different Metallic species and massive organic load into the next-door river systems [1,2]. Not only do these pollutants caused aquatic ecosystem disturbance; but also some of them (pb, cd, cr, etc.) subsequently enter the food chain, and threaten human health by poisoning and accumulating in benthos, aquatic plants and other upper level of animal hierarchy [3- 5].

The Hasdeo River, which is more than 100 km drainage system in human province, India, has been receiving metals along with organic matter from mining, coal, paper industries for last 15 years in korba region, Chhattisgadh, India. Korba is highly industrialized and important area that contributes nationally and globally to the economy of India. As a consequence of this industrialization, CPCB [6] identified Korba as a pollution hub and fifth rank in the critically polluted area. Because of continuous domestic and industrial discharges into Hasdeo River its water quality is deteriorated in that region. So it is necessary to continuously monitor pollution load of the rivers through this future actions can be taken effectively to reduce toxic effects of pollution on living beings. Investigation on water quality assessment of Hasdeo River has been reported earlier [7, 8]. However, there are two critical questions limits the aforementioned research papers: [a] what is the complete physico-chemical texture along with heavy metal in Hasdeo River? [b] How interpret Hasdeo River water quality for different purposes? 

Water quality index (WQI), Heavy metal pollution index (HPI) and metal index (MI) are the mathematical technique used to transform large quantities of water quality parameters into a single number, which provides a simple and understandable tool to interpret quality and possible uses of a water body like drinking, irrigation, fishing. Keeping above problems in our mind the objectives of this study were: 1: assessment of current chemical texture of Hasdeo River water in terms of organic and inorganic load at different location; and 2: Evaluation of water quality in terms of WQI, HPI and MI.

## Materials and Methods

### Study area:

The Hasdeo River, a tributary of Mahanadi River originates from the valley of chota Nagpur, hill region of a Deogarh, Chattishgarh, India and flows through Korba, Janjgir-Champa district and joined in Mahanadi River. The climate in the Korba region is tropical with average temperature 26.6°C and 1420 mm average rainfall. Korba is situated at 22.3595°N, 82.7501°E on the banks of the Confluence of river Hasdeo.

### Water sampling and preservation:

**Figure 1:**
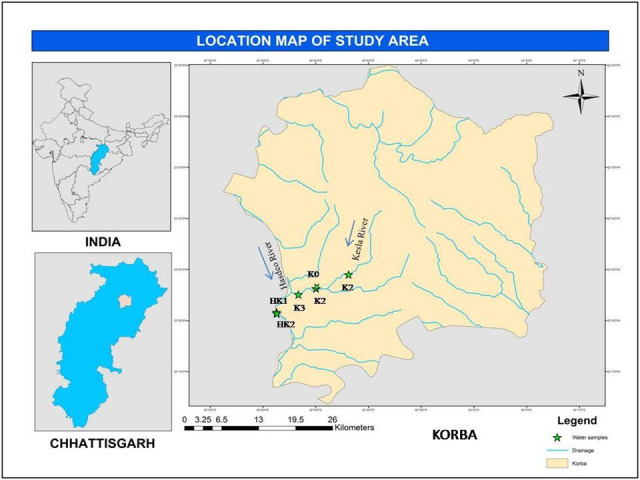
Study sites; K1: Water of Kesla River-Before Mixing With BALCO at Kesla; K0: Waste Water Discharge from BALCO-Industrial Channel Ghat; K2: BALCO Waste Water Mixing with Kesla River; K3: BALCO Waste Water Mixing with Kesla River (At Dengur Nala, 5 Km away from mixing point); HK1: BALCO Waste Water Mixing with Hasdeo River Upstream; HK2: BALCO Waste Water Mixing with Hasdeo River Downstream

Samples were collected from six different stations, 2 from Hasdeo and 4 from Kesla River in Korba (Figure 1). Sampling was done at a depth of 15 cm, below the surface in clean, sterilized capped containers in triplicates from each site. Collected samples were stored in the laboratory at 4°C until processed or analyzed. Water sample Collection, preservation and analysis were performed according to standard protocols of American Public Health Association [9].

### Analytical procedures:

All the physicochemical parameters of river water were analyzed by using the standard protocols of APHA [9]. During sampling of river water temperature was recorded by using Mercury Thermometer. From multiple parameters ion meter (Thermo Orion 5 Star) pH, electrical conductivity (EC), nitrate (NO_-3_), chloride (Cl-) and fluoride (F-) were analyzed. Analysis of sodium and potassium was performed by using flame photometer (CL-378 Elico, India). Sulphate (SO_4_-) and phosphate (PO_4_-) was measured by using double beam UV–Visible spectrophotometer (Perkin Elmer Lambda 35) from turbidimetric and stannous chloride method respectively. Total solid (TS), Total Dissolved Solid (TDS) and Total Suspended Solid (TSS) were measured using gravimetric method. Total Hardness (T- hard as CaCO_3_) was determined by the EDTA titrimetric method. Acid–base titration was used to determine total carbonate and bicarbonate alkalinities. Color was measured through visual comparison method, Chemical Oxygen Demand (COD) through open reflux method and Biological Oxygen Demand (BOD) through 5-day method. Heavy metals: chromium, cadmium, copper, iron, nickel, lead, zinc and manganese were acid digested with nitric/ perchloric acid mixture (5:1) and measured by using Inductively coupled plasma spectrophotometer (ICP) (Thermo Electron; Model IRIS Intrepid II XDL, USA). All observations were recorded in triplicate and their average values are reported.

### Interpretation of results in terms of Water quality index (WQI):

WQI has been calculated from the weighted arithmetic index method in the following steps [10,11]:

Equation →1 (see PDF)

### Calculation of Qi value:

 Equation →2 (see PDF)

Where, Vi = measured value of ith water quality parameter present, Vo = ideal value of ith water quality parameter in pure water, Vo = zero for all parameters except for pH =7.0 and DO = 14.6 mg/l [12]. Si = standard permissible value of ith water quality parameter.

### Calculation of Wi value:

Unit weight (Wi) for various water quality parameters is inversely proportional to the recommended standards for the corresponding water quality parameters.

Equation →3 (see PDF)

Where K is the proportionality constant of the "Weights" for various water quality parameters 

Equation →4 (see PDF)

The water quality has been classified on the basis of WQI into 5 Classes: WQI 0–25 excellent, grade A; WQI 26–50 good, grade B; WQI 51–75 poor, grade C; WQI 76–100 very poor grade D and WQI >100 unfit, grade E.

### Interpretation of results in terms of Metal quality index:

To determine heavy metal contamination in Hasdeo River water two different quality indices are used in this study. Heavy metal pollution index (HPI) is a powerful technique for the assessment of overall water quality with respect to heavy metals [13]. HPI is based on the weighted arithmetic quality mean method. The HPI model is described by Mohan et al. [14].

Equation →5 (see PDF)

Where, Qi = sub index of ith water quality parameter, Wi = unit weightage of ith parameter, n = number of parameters. Wi of ith parameter is defined as inversely proportional to the standard permissible value (Si) for each parameter [14,15].

Calculation of Sub index Qi parameter is given by 

Equation →6 (see PDF)

Where, Mi = measured value of heavy metal of ith parameter present, Ii = ideal value or highest desirable value of ith parameter, Si = standard permissible value of ith parameter, the sign (-) indicates numerical difference of the two values, ignoring the algebraic sign. The critical pollution index value for drinking water is 100.

The metal index (MI) calculates the relative contamination of different heavy metals separately and manifests the summation of generated components as a representative [16] to determine the level of heavy metal contamination of the surface water. With the MI suitability of water for drinking purpose can be interpreted [17] (Caerio et al. 2005). This index can be expressed by the following equation:

Equation →7 (see PDF)

Where MI = metal index, Ci = concentration of each element, MAC = maximum allowed concentration for each element, subscript i = ith sample. The higher value of MI affects water quality more and is more harmful for human health. MI value >1 is concluded as threshold of warning [18]. MI is classified according to Caerio et al. [17]; into six classes: class 1- MI <0.3 very pure; class II- MI 0.3-1.0 Pure; class III- MI 1.0–2.0 Slightly affected; class IV- MI 2.0–4.0 Moderately affected; class V- MI 4.0–6.0 Strongly affected and class VI- MI >6.0 Seriously affected.

### Statistical analysis:

One-way analysis of variance (ANOVA) and Tukey's multiple comparison tests were used to compare the mean values of the different physico-chemical parameters for all the sampling sites and to identify the homogeneous type of the data sets. Pearson correlation matrix was also calculated by the Pearson correlations test for the different physicochemical parameters and heavy metal concentrations of river water from different sampling sites. Statistical analysis was carried out using the statistical package for the social science, version 22 (SPSS-22, IBM, Chicago, USA). P < 0.05 was considered as statistically significant.

## Results and discussion:

### The Physicochemical Characteristics of River Water:

The physicochemical parameters of river water were analyzed statistically and results are given in Table 1.

### Temperature:

Temperature is an essential and changeable environmental factor that affects overall quality of water. During daytime the mean temperature recorded at all sampling sites were in the range of 24.7-25.5 °C. Temperature was almost equal at sampling site K1, K0, K2 and K3 and not showing statistically significant difference (ANOVA/Tukey's t test; P>0.05).

### pH:

pH of all the sampling sites were 7.4 to 8.0, within a range appropriate limits of Bureau of Indian standard [19] for water supply and aquatic life which indicates slightly alkaline nature of river water might be due mixing of urban runoff or industrial wastewater having bicarbonates and carbonates of calcium and magnesium. Site K1, K0, K2 and K3 were homogenate in pH (ANOVA/Tukey's t test; P>0.05).

### Color:

The color of river water was significantly different in sampling sites K2, HK1 and HK2 (ANOVA/Tukey's t test; P>0.05), highest color 112.4±3.33 CoPt (Cobalt Platinum Color Unit) was observed at site K2 might be due to higher accumulation of different organic and inorganic compounds coming from BALCO industry and CSEB fly ash dykes.

### Conductivity:

In this study, almost all the samples had high electrical activity and statistically significant difference among each other (ANOVA, P > 0.001) and out of them site HK2 had highest EC value 1815±27 µS/cm which exceeds the permissible limit (1,000 µS/cm) of BIS, 2005 might be due to urban discharges.

### TDS, TSS and TS:

In a liquid, TDS is defined as a measure of the combined content of all substances i.e. inorganic and organic originating from natural sources, urban discharges, industrial waste water and chemicals used in the water treatment process. The TDS of river water was maximum 1200 mg/L and minimum 202 mg/L at sampling sites HK2 and K1, respectively. These results indicated that water from HK2 was containing approximately 6 fold higher TDS than K1, that might be due to the mixing of pollutants through industrial and domestic activities. TDS from K1, K0, K2 and K3 were within a range of WHO water quality standard that is 500 mg/L and HK1 and HK2 were exceeded through this range. If this water will be used for drinking purposes it may induces an unfavourable physiological reaction in the transient consumer and gastro-intestinal infections [20]. The total suspended solid (TSS) was recorded highest at HK2 (745 mg/L) and lowest at K1 (42 mg/L). Total solids (TS) are a measure of the suspended solids and dissolved solids in water. TS recorded maximum 1945 mg/L and minimum 244 mg/L at HK2 and K1, respectively. TS, TDS and TSS varied drastically among different sampling sites (ANOVA, P < 0.001) except site K0 and HK1 for TSS.

### Dissolved oxygen:

Dissolved oxygen (DO) is an important parameter to access quality of river water [21]. Its deficiency directly affects the river ecosystem due to bioaccumulation and biomagnifications. Pattern of DO level at different sampling sites was K1>K3>K2>K0=HK1>HK1. At K1 site, a negative relationship between DO and BOD as maximum DO and minimum BOD was recorded that is an indication of high re-aeration rate and rapid aerobic oxidation of biological substances which ultimately results good health of water system. A similar pattern was recorded for the river Suswa and other river [22-23]. The difference among sampling sites for DO was of not statistically significant probably it might be due to the turbulences and flow rate of river water at different sampling sites.

### BOD and COD:

High BOD adversely affects the river water quality and biodiversity. In this study, BOD ranged from 7 mg/L (minimum at K1) to 44.4 mg/L (maximum at HK2), which were above the CPCB standards (2 to 3 mg/l for Class A, B and C) [24]. Comparatively, lower BOD at upstream sampling point HK1 than downstream sites HK2 was observed that clearly suggested the mixing of wastewater from the discharge of effluents from city and industries over Hasdeo River at Korba. Similar pattern was recorded for Hindon River at Ghaziabad [23]. The COD values varied from 15 mg/L (K1) to 80 mg/L (HK2). Elevated levels of COD in HK2 indicated poor water quality might be caused by sewage, urban, agricultural and industrial effluents. Site HK1 and HK2 were statistically significant for BOD and HK2 was for COD (ANOVA/Tukey's t test; P<0.05).The sulphate, alkalinity, nitrate and chloride were within the limit of drinking water standards of WHO (Table 2). The phosphate was highest at sampling site HK2 (0.7 mg/L) and lowest at K2 (0.25 mg/L). Total hardness was ranged from 50 mg/L to 470 mg/L, sodium 37 mg/L to 311 mg/L and fluoride 0.28 mg/L to 1.68 mg/L at all the sampling sites. The sampling sites HK2 was exceeded from BIS, 2005 desirable limit (1.0 mg/L) might be due to BALCO industry. BALCO (Bharat Aluminium Company Limited), one of the 4 major primary aluminium producers of India is situated at Korba. Chhattisgarh ranks one of the highest coals producing state in India because of contribution of coalmines located at Korba such as Gevra Area (one of the biggest coal mines of Asia), Kusmunda Area and Dipka Area. This is supported by Ravikumar et al. [25]. However, nitrite was below detectable limit at all sampling sites except HK2 (0.2 mg/L).

### Heavy metals:

The metal analysis of different sites is shown in Table 3/. Accumulation of Cd in the human can lead to kidney, bone and pulmonary damage [26]. In this study, Cd was below detectable level or within a desirable limit (0.01 mg/L) and Cr was within the desirable limit (0.05mg/L) approved by BIS, (2005) at all sites. Sources of Cr in Hasdeo River could be discarded chromium batteries, surface runoffs, and solid waste dump leachates. Iron was exceeded from desirable limit (1 mg/L) approved by WHO or BIS (2005) [19,27], at HK1 and HK2 and rest of sites were within this range. Sources of Fe in river water might be from weathering process of soil formation, industrial effluents, municipal wastewater, leachate from refuse dump sites that are discharged into river water. The concentration of the lead was found to be 2 to 16 times higher comparing to its desirable limit (0.05mg/L) except site HK1 where lead was within a range. One of the major sources of lead is industrial effluent discharged in river water without any prior treatment or improper treatment [28]. The high concentration of lead in river water can damage the central nervous system, kidneys and blood system [29]. The concentration of Mn is highest at K3 (0.195 mg/L) and lowest at K1 (0.4 mg/L). Water containing excessive level of Mn may leads to objectionable staining on cloth washing.

### Water Quality Index of the river water:

**Figure 2:**
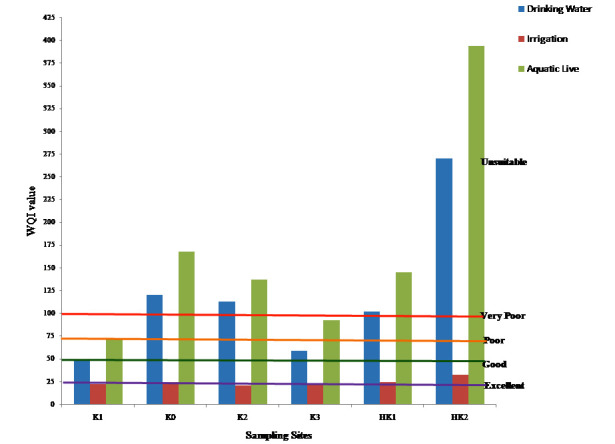
Water quality index of different sampling sites of Hasdeo River water for drinking, irrigation and aquatic live utilization.

Figure 2 shows the values of the WQI of Hasdeo River at different sampling site. Using guidelines of BIS, 2005 and WHO compute WQI score for drinking water usage, 2011. Guidelines of FAO, 1994 and CCME, 2007 are used to compute the WQI score for irrigation and Protection of aquatic life respectively. 13, 8 and 9 variables have been used to calculate WQI for dinking, irrigation and aquatic life criteria, respectively. The results showed that variables Nitrite, Fluoride and BOD for drinking water, phosphate, pH and nitrate for irrigation and nitrate, DO and COD for aquatic live has key significance in water quality assessment because of their high weight (Wi) (Table 2). In this study WQI score for drinking water usage was ranged 47.33 to 269.68 indicated water quality good to unsuitable. Water quality for irrigation and aquatic lives was excellent to good (WQI 20.69 - 32.25) and poor to unsuitable (WQI 71.82- 393.25) respectively (Figure 2). On the basis of computed WQI site HK2 was most polluted site unsuitable for drinking and aquatic live and good for irrigation might be due to high domestic sewage disposal and industrial activities.

### Heavy Metal Pollution Index (HPI):

Calculations and result of HPI for all the sampling sites with unit weightage (Wi) and standard permissible value (Si) is shown in Table 3. In this study HPI ranged 75.34-237.19 (Table 4). All the sampling sites except site K3 and HK1 had HPI values above 100 which is the critical pollution index value above which the overall pollution level should be considered unacceptable [13]. The calculated mean HPI of Hasdeo river water is 130.19 that are above the critical index value 100. Percentage deviation from the mean HPI showed that site K1, K3 and HK1 had percentage deviation on the negative side, which is an indication of a slightly better quality of water with respect to heavy metals. Zn and Cu were not much contributed in evaluation of HPI of Hasdeo River because of less weightage (Wi) values respectively. Heavy metals like Cd, Ni, Pb, Cr, Fe and Mn had high weightage (Wi) values that gave high HPI values indicating that smaller concentration of these heavy metals in river water contributes in poor water quality (Table 3). Overall, the Hasdeo river water with respect to heavy metals contamination is a serious issue among all the sampling sites except site K3 and HK1. It might be due to industrial, agricultural and domestic activities.

### Metal index (MI):

Metal index calculations and results are shown in Table 4. According to metal index values, all the sampling sites were seriously affected with metal pollution and classified as class VI. MI reached 25 at Site HK1 and 100 at site HK2 respectively.

### Correlation Analysis:

Correlation analysis for 20 physico-chemical parameters and 8 heavy metals from different sampling sites of Hasdeo River were performed (Table 5). Correlation coefficient (r) is defined as statistical measurement of the interdependence of two or more random variables. Correlation analysis measures the closeness and degree of linear association between independent and dependent variables [30]. In this study EC, TDS, BOD, COD and Total Hardness values were strongly correlated with each other because EC mainly depends on total ionic content or dissolved inorganic substance. EC can be used to rough estimate the total dissolved solid (TDS) in water as TDS increases with increase in EC (dissolved ions concentration). However, EC also exhibited good significant positive correlation with F, Cl, alkalinity, nitrate and potassium (r2>0.944). On other hand, pH and dissolve oxygen showed negative correlation with most of the physico-chemical parameters. In this study, Heavy metals were not showing significant correlation among them (data not shown).

### Conclusions:

Water quality assessment of the river using WQI, HPI and MI calculations shows that the water of Hasdeo and their tributary river is suitable for irrigation purpose but not for drinking as well as aquatic life.

## Figures and Tables

**Table 1:** Chemical texture of collected river water from different sampling sites

Characteristics	Site K1 (Mean±SD)	Site K0 (Mean±SD)	Site K2 (Mean±SD)	Site K3 (Mean±SD)	Site HK1 (Mean±SD)	Site HK2 (Mean±SD)
pH	8.08a±0.12	8.0a ±0.1	7.99a±0.2	7.92a ±0.1	7.65c ±0.2	7.48c±0.3
Temperature (°C)	24.8a	24.8a	24.7a	25a	25.5c	25.4c
Conductivity (µS/cm)	299.7c ±17.1	712c ±31	606c ±26	511.5c ±20	970c ±21	1815c ±27
Color (Copt.)	81.22a±4.8	81.39a±8	112.4c±7	81.36a±5.3	73.64c±2.8	72.1c ±3.2
TS	244c±14	585c±22	455c ±18	405c ±19	760c ±77	1945c ±63
TDS	202c±7.8	470c ±22.2	400c ±33	340c±15.7	640c±29.9	1200c±53
TSS	42c±1.11	115a±4.2	55c±1.7	65c±1.6	120a±2.22	745c±37
COD	15a±1.7	39b±2.5	33b±1.3	19a±2	30b±2.9	80c±3
BOD	7a±1	19b±1.6	17b±1	9a±1	14.7c±1.8	44.4c±3
Sulphate	44.42b±3.2	31.6c±1.5	44.56b±5	62.16c ±1.8	43.02b±1.7	104c±4.6
Phosphate	0.28a±0.01	0.34b±0.2	0.25a±0.06	0.34b±0.04	0.45c±0.09	0.7c±0.03
DO	9.8a±0.23	7.3b±0.15	7.4b±0.53	8.7ab±0.82	7.33b±0.2	6.27b±0.6
Total hardness	50c±1.1	164c±3.6	124c±4.1	82c±2.3	146c±4.7	470c±18
Alkalinity	26c±1.6	82a±2.4	61c±1.9	41c±1.7	78a±2.6	124c±3.9
Chloride	23a±1.1	39b±2.6	35bd±0.9	26a±0.4	33d±0.6	66c±2.8
Fluoride	0.28a±0.09	0.8b±0.05	0.86b±0.06	0.34a±0.03	0.84b±0.05	1.68c±0.1
Nitrate	0.24c±0.01	0.9a±0.01	0.88a±0.05	0.37c±0.02	0.87a±0.03	1.9c±0.01
Nitrite	BDL	BDL	BDL	BDL	BDL	0.2±0.02
Na	37.19c±1.9	77.11c±5	72.07c±3	66.47c±1.8	88.10c±3.2	311c±18
K	2.09a±0.05	3.1a±0.08	2.69a±0.01	2.62a±0.04	4.93c±0.02	8.83c±0.7
All the parameters are in mg/L except colour, temperature, conductivity and pH; BDL: Bellow Detection Limit; SD standard deviation. The mean value of each parameter and sampling site with different Superscripts (lowercase letters) are significantly different. (ANOVA; Tukey's t test, P < 0.05).

**Table 2:** Guidelines of water quality parameters for drinking, irrigation and aquatic live and respective Wi computations for WQI.

Parameters	Drinking Water		Irrigation		Aquatic Live	
	WHO/BIS (Si)	Unit weight (Wi)	FAO (Si)	Unit weight (Wi)	CCME (Si)	Unit weight (Wi)
pH	8.5a	0.0401	8.5	0.163	9	0.1217
Temperature	-	-	-	-	28	0.039
Conductivity	1000b	0.0003	3000	0.0005	-	-
TDS	500 a	0.0007	2000	0.0007	500	0.0022
TSS	-	-	-	-	25	0.0438
COD	10a	0.0341	-	-	7	0.1564
BOD	3b	0.1136	-	-	-	-
Sulphate	250a	0.0014	960	0.0014	-	-
Phosphate	-	-	2	0.693	-	-
DO	6b	0.0568	-	-	5.5	0.1991
TH	500a	0.0007	-	-	-	-
Alkalinity	250a	0.0014	-	-	20	0.0548
Chloride	200a	0.0017	1063	0.0013	120	0.0091
Fluoride	1b	0.3408	-	-		
Nitrate	11a	0.031	10	0.1386	2.93	0.3738
Nitrite	0.9a	0.3787	-	-	-	-
Na	-	-	919	0.0015	-	-
Total		1.0012		1		1
a = WHO (2011); b = BIS (2005); FAO (1994); CCME, 2007; All the parameters in mg/L except colour, temperature, conductivity and pH.

**Table 3:** Standard values, ideal values and weightage of metals in the study area.

Heavy metals	Standard PV (Si)	Highest DV (ppb) (Ii)	Measured Value (Mi or Ci)						Unit	MAC (ppb)
			Site K1	Site K0	Site K2	Site K3	Site HK1	Site HK2	weightage (Wi)	
Cd	10	0	10	BDL	BDL	BDL	10	11	0.1	3
Cr	50	0	184	170	113	102	64	53	0.02	50
Cu	1500	50	13	8	22	32	19	101	0.0006667	1000
Fe	1000	300	776	893	542	526	2089	2189	0.001	200
Mn	300	100	45	88	63	195	194	56	0.0033333	50
Ni	20	20	89	29	40	30	44	26	0.05	20
Pb	50	0	131	494	550	259	43	820	0.02	10
Zn	15000	5000	54	66	103	115	96	86	0.0000667	5000

**Table 4:** Heavy metal pollution index (HPI) and Metal Index (MI) of various sampling sites of Hasdeo River

Sampling Sites	HPI			MI		
	HPI value	% Deviation from Mean HPI	Interpretation	MI value	Interpretation	Grading
K1	116.7	-10.36	Critically contaminated	29.3671	Strongly Affected	V
K0	136.72	5.0164	Critically contaminated	60.4962	Strongly Affected	V
K2	136.47	4.8229	Critically contaminated	63.2726	Strongly Affected	V
K3	78.72	-39.54	High metal pollution	36.025	Strongly Affected	V
HK1	75.34	-42.13	High metal pollution	25.4765	Strongly Affected	V
HK2	237.2	82.19	Critically contaminated	100.2099	Strongly Affected	V

**Table 5:** Correlation matrix of physico-chemical analysis.

	pH	Temp	Cond	Color	TS	TDS	TSS	COD	BOD	Sul	Phos	DO	TH	Alk	Cl	F	NO3-	NO2-	Na	K
pH	1																			
Temp	-0.921	1																		
Cond	-0.929	0.731	1																	
Color	0.535	-0.697	-0.431	1																
TS	-0.893	0.677	0.99	-0.44	1															
TDS	-0.929	0.731	1	-0.432	0.99	1														
TSS	-0.821	0.587	0.947	-0.437	0.983	0.948	1													
COD	-0.768	0.484	0.95	-0.264	0.961	0.95	0.948	1												
BOD	-0.773	0.486	0.951	-0.258	0.967	0.951	0.959	0.999	1											
Sul	-0.742	0.536	0.792	-0.359	0.85	0.794	0.898	0.757	0.784	1										
Phos	-0.94	0.813	0.962	-0.637	0.966	0.963	0.943	0.868	0.873	0.836	1									
DO	0.717	-0.486	-0.829	0.056	-0.765	-0.827	-0.664	-0.839	-0.82	-0.421	-0.666	1								
TH	-0.827	0.571	0.973	-0.363	0.99	0.973	0.982	0.989	0.992	0.827	0.926	-0.789	1							
Alk	-0.816	0.598	0.944	-0.322	0.911	0.943	0.843	0.945	0.93	0.585	0.848	-0.941	0.924	1						
Cl	-0.769	0.484	0.95	-0.264	0.962	0.95	0.948	1	0.999	0.758	0.868	-0.84	0.989	0.945	1					
F	-0.807	0.539	0.952	-0.177	0.937	0.951	0.891	0.974	0.971	0.679	0.837	-0.907	0.954	0.962	0.973	1				
NO3-	-0.807	0.537	0.959	-0.21	0.947	0.958	0.905	0.985	0.981	0.696	0.851	-0.906	0.967	0.971	0.984	0.998	1			
NO2-	-0.777	0.53	0.91	-0.387	0.96	0.912	0.993	0.924	0.94	0.926	0.91	-0.601	0.961	0.784	0.924	0.858	0.872	1		
Na	-0.845	0.6	0.964	-0.385	0.99	0.965	0.995	0.96	0.97	0.9	0.941	-0.718	0.989	0.868	0.96	0.917	0.929	0.986	1	
K	-0.947	0.777	0.991	-0.499	0.988	0.991	0.954	0.917	0.923	0.821	0.982	-0.752	0.957	0.897	0.917	0.915	0.92	0.923	0.962	1
Temp: temperature; Cond: conductivity; TS: Total solid; TDS: total dissolve solid; TSS: total suspended solid; Sul: Sulphate; Phos: phosphate; DO: dissolve oxygen; TH: total hardness; Alk: alkalinity; Cl: chloride; F: Floride; Na: sodium; K: potassium
